# Diagnostic Approach to Congenital Cystic Masses of the Neck from a Clinical and Pathological Perspective

**DOI:** 10.3390/dermatopathology8030039

**Published:** 2021-08-01

**Authors:** Amanda Fanous, Guillaume Morcrette, Monique Fabre, Vincent Couloigner, Louise Galmiche-Rolland

**Affiliations:** 1 Pediatric Otolaryngology-Head and Neck Surgery, AP-HP, Hôpital Universitaire Necker Enfants Malades, 75015 Paris, France; amandafanous@gmail.com (A.F.); vincent.couloigner@aphp.fr (V.C.); 2 Department of Pediatric Pathology, AP-HP, Hôpital Robert Debré, 75019 Paris, France; guillaume.morcrette@inserm.fr; 3 Department of Pathology, AP-HP, Hôpital Universitaire Necker Enfants Malades, Université Paris Descartes, 75015 Paris, France; mofabre@gmail.com; 4 Faculté de Médecine, Université de Paris, 75015 Paris, France; 5 Department of Pathology, University Hospital of Nantes, 44000 Nantes, France

**Keywords:** congenital cystic mass, pediatrics, cervical cyst, cervical malformation

## Abstract

Background: neck cysts are frequently encountered in pediatric medicine and can present a diagnostic dilemma for clinicians and pathologists. Several clinical items enable to subclassify neck cyst as age at presentation, anatomical location, including compartments and fascia of the neck, and radiological presentation. Summary: this review will briefly describe the clinical, imaging, pathological and management features of (I) congenital and developmental pathologies, including thyroglossal duct cyst, branchial cleft cysts, dermoid cyst, thymic cyst, and ectopic thymus; (II) vascular malformations, including lymphangioma. Key Messages: pathologists should be familiar with the diagnostic features and clinicopathologic entities of these neck lesions in order to correctly diagnose them and to provide proper clinical management.

## 1. Introduction

Congenital cystic masses of the pediatric neck can be broadly divided into medial and lateral lesions [1]. Medial lesions include thyroglossal duct cysts, dermoid cysts and bronchogenic cysts. Lateral lesions include branchial cleft cysts, lymphangiomas and thymic cysts. As most congenital lesions manifest during infancy and early childhood, the patient’s age provides important diagnostic information. A painless soft or fluctuant cervical mass is the first clinical manifestation in most cases. Following physical examination, ultrasonography (US) is usually performed, defining the size and extent of the mass, demonstrating its relationship to surrounding normal structures, and confirming the cystic nature of the lesion. Computed tomography (CT) and magnetic resonance imaging (MRI) can then provide additional useful clinical information [2].

The aim of this review is to briefly describe each of the above entities in order to provide the clinician and the pathologist with the relevant clinical and pathological characteristics.

## 2. Details and Discussion

The range of pathology seen in the neck region is very wide and to a large extent probably mirrors the complex signaling pathways and careful orchestration of events occurring during the development of this region. As is true of pediatric pathology on general pathology, within this age group is as diverse as its adult counterpart. Cases that come across the pediatric neck surgical pathology bench are more heavily weighted toward developmental and congenital lesions.

We report on the clinical presentation, diagnostic evaluation and therapeutic management of cystic lesions of the neck. Congenital cystic masses of the neck include thyroglossal duct cysts, branchial cleft cysts, lymphangioma, dermoid/epidermoid cysts, thymic and bronchogenic cysts (visceral cysts). These lesions vary in prevalence from common (thyroglossal duct cysts, branchial cleft cysts, and lymphangioma) to very rare (thymic cysts and cervical bronchogenic cysts). The vast majority of these cysts are benign in nature, unlike what is observed in adults.

The evaluation of a patient suspected of having a congenital cervical cystic mass should follow an orderly progression [3]. The clinical history and physical examination of the patient are the most important factors in the evaluation of a congenital neck mass. An appropriate knowledge of the embryology and anatomy of the cervical region frequently allows the differential diagnosis to be narrowed.

**Figure 1:**
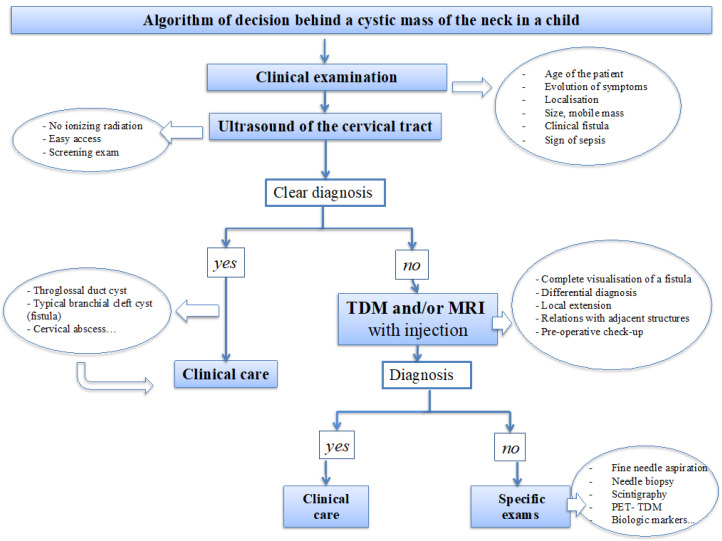
Diagnostic algorithm with the different steps depending on clinical examination and imaging.

An algorithm of the decision behind cervical cysts in the pediatric population is proposed (Figure 1).

The patient’s age and location of the cyst provide important diagnostic information. A painless soft or fluctuant cervical mass is the first clinical manifestation in most cases. These lesions are usually slow-growing masses and typically cause symptoms due to enlargement or infection. Congenital ‘‘benign’’ lesions can sometimes cause significant morbidity and even mortality if they compress the airway or other vital structures.

Following physical examination, ultrasonography (US) is the most frequently used imaging modality due to its accessibility and absence of ionizing radiation. US helps to define the size and extent of the mass, demonstrates its relationship to surrounding normal structures, and confirms the cystic nature of the lesion. US may be sufficient if diagnosis is clear and in adequation with clinical presentation, typically for most thyroglossal duct cyst and branchial cleft cysts with cutaneous fistulization. Computed tomography (CT) also provides this information and is ideally suited for the evaluation of soft tissue adjacent to larger masses that cannot be entirely visualized with US. Moreover, CT is superior for detecting calcification and, when contrast material is administered, the vascularity of lesions. Magnetic resonance (mass MR) imaging, with its multiplanar capability and superior contrast resolution, demonstrates the full extent of the mass and gives important additional information for accurate preoperative planning. This can be especially relevant in cases of extension into the mediastinum or deep spaces of the neck. Furthermore, MR imaging offers superior resolution for evaluating masses located in anatomically complex areas, such as the floor of the mouth [2].

Malignant neoplastic lesions originating from these cervical cysts are very rare and arise in association with a heterotopic tissue. However, despite the rarity of malignant cystic cervical masses in children, it remains crucial to remain very careful with differential diagnosis. One should always suspect malignancy behind heterogeneous mass with solid and cystic components or equivocal clinical and radiological presentation [4]. 

The purpose of this review is to present the clinical, pathological, and radiological features of the most common congenital cystic lesions of the neck, emphasizing their embryologic origin, and the differential diagnosis.

We will discuss the details of the different congenital cystic lesions of the neck, emphasizing their embryologic origin, the clinical and radiologic findings, the gross and histopathologic features, the differential diagnosis, and their management. All these items are summed up in Table 1 and Table 2.

### 2.1. Thyroglossal Duct Cyst

In the embryo, the primitive thyroid gland begins as a ventral diverticulum arising in the floor of the embryologic pharynx, between the tuberculum impar and the copula, which later forms the foramen cecum located at the base of the tongue. The thyroid then descends caudally in close relation to the hyoid bone before reaching its final position in the midline of the neck below the level of the cricoid cartilage. The resulting thyroglossal duct tract normally atrophies by the 10th week of gestation. Thyroglossal duct cysts result from failure of the complete obliteration of the thyroglossal duct tract, and can be located anywhere along the descent of the thyroid gland.

#### 2.1.1. Clinical Findings

**Figure 2:**
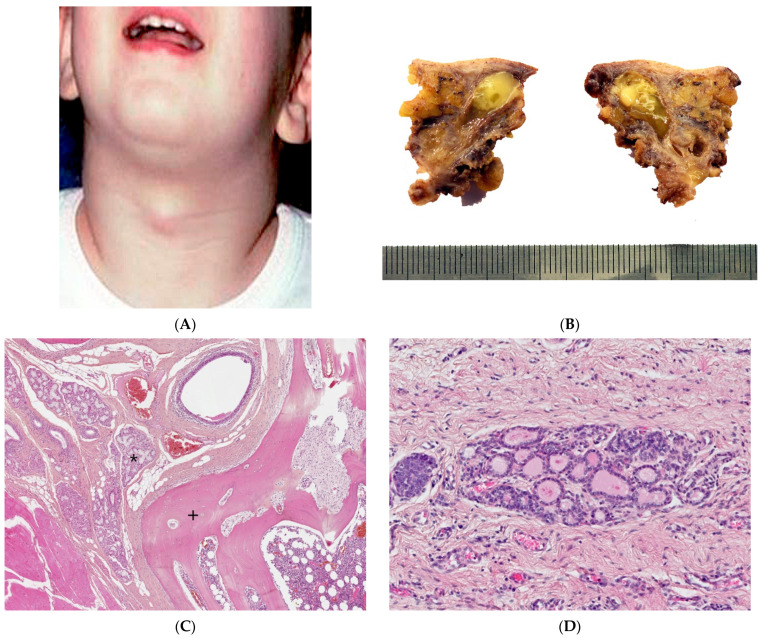
Thyroglossal duct cyst. (**A**) Clinical examination: typical picture of a non-infected thyroglossal duct cyst presenting as a midline neck mass. (**B**) Gross examination: cystic lumen filled with gelatinous and inflammatory material. (**C**) (H.E.S). Small cystic structure lined by ciliated pseudostratified columnar epithelium; mucous glands (*) on the left; hyoid bone (+) on the right, in close relation to the cyst. (**D**) (H.E.S), ectopic thyroid follicles in the cystic wall.

A thyroglossal duct cyst is the most common congenital neck mass and the second most common of all childhood cervical neck masses. Most are diagnosed at or before 10 years of age. They can occur anywhere along the thyroglossal duct tract but are most commonly found below the hyoid bone, displaying upwards displacement with tongue protrusion and swallowing. The cysts are usually a few centimeters in diameter and are round and smooth on palpation (Figure 2A).

Thyroglossal duct cysts are often asymptomatic. If very large, dysphagia may be present. Infection may occur, often in conjunction with upper respiratory tract infections, and can cause painful cystic enlargement, abscess development or rupture with cutaneous sinus formation. 

The main clinical differential diagnosis includes dermoid cyst, ectopic or lingual thyroid and lymphadenopathy. Ultrasonography is the first line radiological assessment tool, followed by magnetic resonance imaging (MRI).

Heterotopia-associated thyroid carcinoma, most often papillary thyroid carcinoma, may extremely rarely arise in thyroglossal duct cysts, with 27 case reports at a mean age of 12 years of age. The prognosis remains excellent. All carcinomas have been discovered after surgical excision.

#### 2.1.2. Gross and Histopathological Features

A thyroglossal duct cyst usually corresponds to a single cyst, typically less than 2 cm in size, filled with mucoid, squamous or purulent material (Figure 2B). It may also be composed of several smaller cysts that are sometimes difficult to identify macroscopically. Occasionally, when inflammatory processes are prominent, no residual cystic space may be clearly identified.

In non-inflamed cysts, the cyst lining is ciliated pseudostratified columnar and/or squamous epithelium. Small cystic structures might only be noted in close relation to the hyoid bone, which must be sampled (Figure 2C). Ectopic normal thyroid tissue in the cyst wall can be found in 50 to 70% of cases, varying with sampling by the pathologist (Figure 2D). Inflammation and foreign body giant cell reaction are commonly noted. Mucous glands may also be present. C-cells are not present in thyroglossal duct cysts due to the different embryologic origin [5,6,7].

#### 2.1.3. Brief Account of Patient Management

The recommended treatment for thyroglossal duct cysts is a Sistrunk procedure, which entails complete resection of the cyst, the entire remnant thyroglossal tract stalk, and the body of the hyoid bone. Occasionally, an extension to the tongue base requiring a concomitant intra-oral surgical approach is present. This is the only accepted surgical procedure to limit complications (1%) and recurrence (3%).

### 2.2. Branchial Cleft Cysts

Branchial cleft cysts are the second most common congenital neck masses in the pediatric population. Branchial cleft anomalies are congenital malformations related to embryological developmental alterations of the branchial apparatus resulting in cysts (no opening), sinuses (single opening to skin or digestive tract) or fistulas (opening to both skin and digestive tract) [8].

The branchial apparatus first appears in the developing embryo at 4 weeks. It consists of six paired mesodermal arches, separated internally by endoderm making up the four pharyngeal pouches, and externally by ectoderm making up the four branchial clefts. The first four arches proliferate, and the last two become rudimentary. Each arch and cleft give rise to its own cartilaginous, muscular, vascular, glandular, and neural components [9].

Branchial cleft cysts are clinically divided into first, second, third or fourth branchial cleft cysts, depending on the anatomical location of the lesion. This stratification corresponds to the arch involved in pathogenesis. These cysts are closed structures lined by squamous epithelium.

The first branchial apparatus normally gives rise to the eustachian tube, the tympanic cavity, the mastoid antrum, a portion of the tympanic membrane, the external auditory canal (EAC), the mandible, the maxilla, and the mall eus and incus. For this reason, first branchial cleft cysts, which represent the second most common branchial cleft anomaly accounting for less than 10% of all branchial cleft defects, can arise anywhere from the external auditory canal through the parotid gland to the submandibular triangle [10].

**Figure 3:**
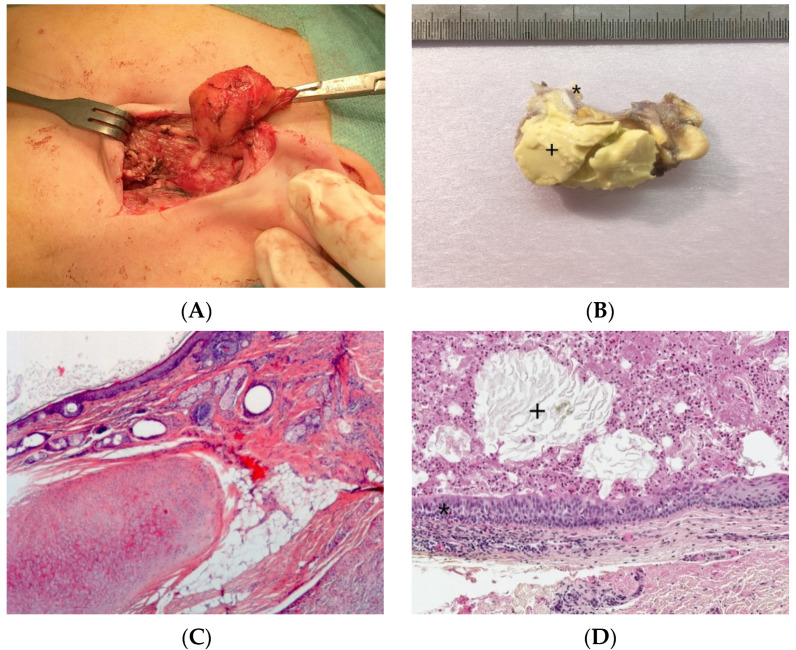

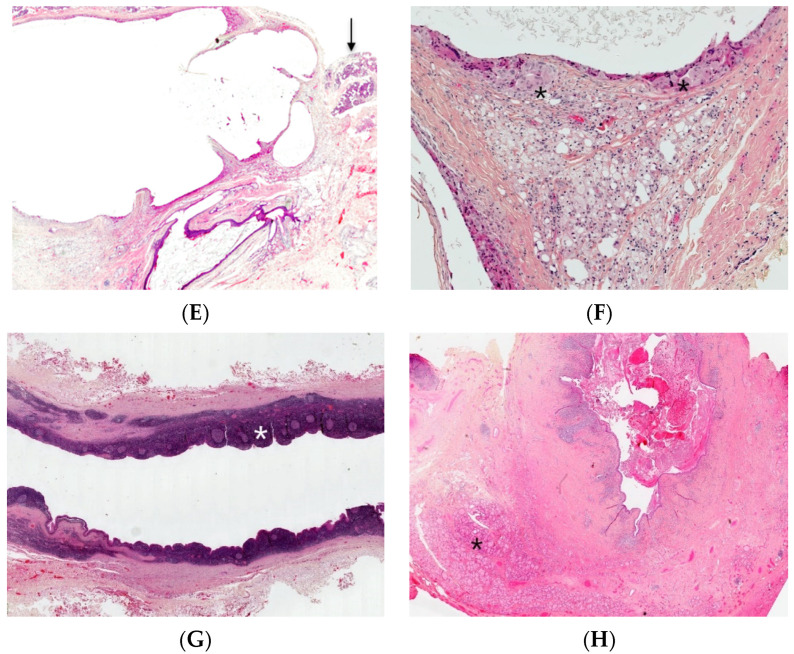
Branchial cleft cysts. First branchial cleft cyst (**A**), operating view. Periauricular type I cyst (**B**), gross examination. Cartilage (*) and necrosis (+) are visible. Periparotid type II with inflammatory changes (**C**) (H.E.S). lined by squamous epithelium and containing cartilage (**D**) (H.E.S). Lined by squamous epithelium and ciliated columnar epithelium (*). The lumen contains macrophages and squamous debris (+) (**E**) (H.E.S), located within the parotid gland (at the upper part, arrow), mainly denuded due to inflammatory changes (**F**) (H.E.S). The epithelial border is mainly replaced by foreign body giant cells (*). Lipid-laden macrophages are observed in the cyst wall and squamous debris within the cyst lumen. Second branchial fistula (**G**) (H.E.S), lined by respiratory epithelium and confluent lymphoid follicles (*). Fourth branchial fistula (**H**) (H.E.S). The wall of the fistula contains thyroid tissue (*).

Two types of first branchial cleft cysts have been described: type I (Figure 3A), periauricular, of ectodermal origin, corresponding to a duplicated EAC and type II (Figure 3B), periparotid, of both ectodermal and mesodermal origin, containing skin and cartilage. An important anatomical difference of clinical and surgical importance is that the cystic tract of type I cysts passes lateral (superior) to the seventh cranial nerve, whereas in type II cysts the tract passes medial (inferior) to the seventh cranial nerve.

Lesions of the second branchial arch are by far the most common branchial anomaly, accounting for 67–93% of cases. Clinically, these cysts are present in the lateral neck, along the anterior border of the sternocleidomastoid muscle. The fistula pathway begins in the lateral neck, runs in between the external and internal carotid arteries, deep to cranial nerve 7 and superficial to cranial nerves 9 and 12, with an internal opening at the level of the oropharynx [11].

Third/fourth branchial cleft anomalies are excessively rare, rendering distinction between these two identities challenging. Anomalies usually manifest themselves as a sinus tract rather than a cyst or fistula. The cysts appear in the lower neck, predominantly left-sided due to the asymmetric development of the vascular structures (on the left, the aortic arch, and on the right, the proximal subclavian artery). Sinuses and fistulas have an internal opening at the level of the piriform sinus [12,13].

#### 2.2.1. Clinical Findings

Classical branchial anomalies are usually isolated. Cysts present as nontender, fluctuant masses, which may become infected and lead to abscess formation. Sinuses and fistulas may be associated with discharge of mucoid and/or purulent secretions from the internal and/or external tract opening.

Differential diagnosis may be challenging between infected branchial cyst and a cervical lymphadenopathy. Anatomical localization should be carefully detailed. Moreover, recurrent or persistent cervical “nodal” mass should raise the question of an underneath branchial cleft cyst and US is usually helpful to clarify the diagnosis.

Precise topographic anatomical assessment allows to define which branchial arch is involved and therefore to diagnose the cyst type. As previously mentioned, first branchial cleft cysts are present in the preauricular or submandibular region, second branchial cleft cysts along the anterior border of the sternocleidomastoid muscle, and third/fourth branchial cleft cysts in the lower neck. Given the close association between third/fourth branchial cleft anomalies and the thyroid gland, an infection can present itself as acute suppurative thyroiditis.

In terms of imaging, US is the initial investigation of choice. CT scan and MRI scan can also aid in diagnosis and precise surgical mapping. When uncomplicated, they are typically visualized as well delineated cystic structures appearing centrally hypoechoic on US with posterior acoustic enhancement. Their US appearances can vary, however, sometimes appearing multilocular with septations, inhomogeneous or even solid. They are uniformly hypodense on CT and can demonstrate the ‘notch’ or ‘beak sign’ between the internal and external carotid arteries. MRI can help in determining the presence and path of sinus formation and presence of inflammatory changes within the adjacent soft tissues. Bilaterality is rare (only 2% to 3%) and often associated with familial predisposition [14].

Branchio-oto-renal syndrome (BOR) may be suspected based on family history, or when second branchial arch anomalies are clinically associated with deafness, pre-auricular pits, auricular malformation and/or renal anomalies (malformations or hypoplasia). If suspected, audiology workup and renal US should be performed. BOR syndrome is inherited in an autosomal dominant pattern, with a prevalence of 1/40,000 in Western countries and results from a mutation in the *EYA1* gene [15,16].

#### 2.2.2. Histopathological Features

Branchial cleft cysts are usually lined by squamous epithelium (90%) or ciliated columnar epithelium (8%) and rarely by both types of epithelia (2%) (Figure 3C,D). Occasionally, the cyst is denudated (Figure 3E) or lined by cholesterol clefts with a foreign body reaction (Figure 3F) due to inflammatory changes. Lymphoid tissue is generally identified (Figure 3G). Salivary tissue, sebaceous glands as well as thyroid tissue may be present (Figure 3H). An ectopic or undescended parathyroid gland may be associated with third/fourth branchial cleft anomalies, since parathyroid glands embryologically arise from these branchial arches. Parathyroid adenomas have also been reported in association with branchial cleft cysts.

#### 2.2.3. Brief Account of Patient Management

Complete surgical excision is undertaken in order to avoid infection and prevent recurrence. This includes a complete excision of the cyst as well as any sinuses or fistulous tracts. Given the various communication patterns of these anomalies, a combined approach is commonly used including a mix of endaural, trans-cervical, trans-parotid and intra-oral/intra-laryngeal (endoscopic) routes.

### 2.3. Lymphatic Malformations

Lymphatic malformations are thought to arise from the early sequestration of embryonic lymphatic channels. There are three main types of lymphatic malformations: microcystic (<2 mL, occurring mostly above the level of the mylohyoid muscle, commonly involving the oral cavity), macrocystic (≥1 cyst, ≥2 mL in size, occurring below the level of the mylohyoid muscle) and mixed (by far the most common, also referred to as lymphangioma, formerly called cystic hygroma). Lymphatic malformations constitute about 5% of all benign tumors of infancy and childhood. The sequestration of lymphatic channels occurs more commonly in the developing jugular lymph sac pair than in the other four embryonic sites of lymphatic system development. From this location, the sequestered site follows the path of the surrounding mesenchyme destined for either the neck or the developing mediastinum. Therefore, these lesions tend to occur in the neck, axilla, and upper mediastinum.

#### 2.3.1. Clinical Findings

The overwhelming majority (about 80–90%) are diagnosed by the time the patient turns 2 years old, the age of greatest lymphatic growth. No gender predilection is reported. Approximately 75–80% of lymphatic malformations involve the neck and the lower third of the face. In children, the most common location is the posterior cervical space, followed by the oral cavity.

These lesions are characteristically infiltrative in nature. Consequently, they may extend inferiorly from the posterior cervical triangle into the axilla and mediastinum or anteriorly into the floor of the mouth and the tongue. If the mass is very large, it may extend across the midline. Only 3–10% of cervical lymphatic malformations are associated with extension into the mediastinum.

Most patients are clinically asymptomatic. The lesion presents itself as a soft, painless and compressible mass in the neck. Size is extremely variable. Very large masses may cause dysphagia or dyspnea by extrinsic compression. The lesion may become painful or enlarge rapidly from acute spontaneous hemorrhage or infection. Disfigurement may also be a significant concern.

They may be associated with chromosomal defects, such as Turner’s syndrome, Trisomy 13, 18 and 21 as well as Noonan syndrome. Additional investigations by means of amniocentesis are therefore indicated when detected on antenatal US.

#### 2.3.2. Histopathological Features

**Figure 4:**
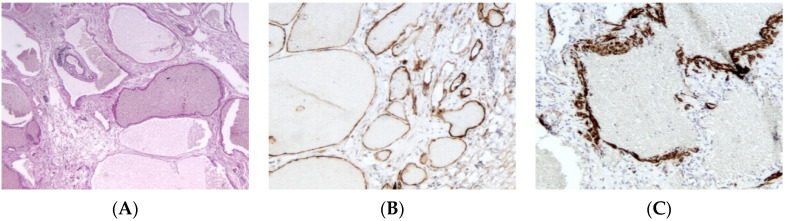
Lymphatic malformation. (**A**), (H.E.S). Collection of large interconnected lymphatic cisterns. Small lymphoid aggregates are also present (at the upper left quarter). (**B**), (D2-40). Positive cytoplasmic immunostaining of endothelial cells. (**C**), (smooth muscle actin). Positive cytoplasmic immunostaining of disorganized smooth muscle in the wall of larger channels.

Lymphatic malformations are composed of multiple dilated cystic spaces separated by minimal intervening stroma (Figure 4A). The cysts vary in diameter from a few millimeters to more than 10 cm, and most contain chylous fluid, a variable number of lymphocytes and/or macrophages, and occasional erythrocytes. These thin-walled spaces are lined by endothelial cells and supporting connective tissue stroma. Focally disorganized smooth muscle is present in the wall of larger channels. Lesions may extend into adjacent soft tissues, invade into muscle, and surround vascular structures.

The macrocystic type is defined by collections of large interconnected lymphatic cisterns; some vessels may have a relatively thick but incomplete smooth muscular lining. Mural lymphoid aggregates are commonly present and help distinguish these lesions from venous malformations.

The microcystic type is comprised of smaller lymphatic channels that may interdigitate between local tissue elements. In superficial lesions, these often protrude into and expand dermal or mucosal papillae, forming lymph-filled vesicles.

An immunohistochemical panel consisting of D2-40 (more consistent staining of small versus large lymphatic channels), Prospero-related homeobox gene-1 (*Prox-1*), vascular endothelial growth factor receptor 3 (VEGFR3), CD31, and CD34 antibodies allows the differentiation of lymphatic malformations from venous ones in pathologic practice (Figure 4B,C) [17,18].

#### 2.3.3. Brief Account of Patient Management

MRI is the diagnostic investigation of choice. If the lesion is asymptomatic, watchful waiting for spontaneous regression may be considered. Medical treatment includes antibiotics if the cyst is superinfected and corticosteroids as needed. Non-surgical treatment remedies include sclerotherapy (with either alcohol, bleomycin or picibanil), radio-frequency ablation or laser therapy. Surgical resection may be considered as a last resort for macrocystic lesions but may be the only viable option for microcystic lesions given that the cysts are too small for sclerotherapy. PIK3CA is known to play a role in regulating cell growth by signaling through the PI3K/mTOR pathway. Medical therapy with the drugs sirolimus or sildenafil can be used to treat both localized and diffuse lymphatic malformations [19].

### 2.4. Dermoid Cyst

Dermoid cysts are congenital masses that result from the sequestration of cutaneous tissues along embryonal fusion lines. They are composed of ectodermal and mesodermal elements only, including epidermal appendages such as hair follicles and sebaceous glands. This is in contrast to teratoid cyst, which are composed of all three embryonic layers (endodermal derivatives, e.g., gastrointestinal or respiratory mucosa or smooth muscle), and epidermoid cysts, composed of ectodermal elements only (without epidermal appendages).

#### 2.4.1. Clinical Findings

Dermoid cysts are present at birth and may grow slowly thereafter or remain stable in size. Most cysts are subcutaneous and recognized in children younger than 5 years old. Dermoid cysts may occur anywhere in the body, with 50% presenting in the head and neck area (30% face and 20% neck). Nasal and palpebral sites are most common in the face. In the neck, the midline location is very characteristic of the diagnosis and may be associated with a tuft of hair. Diagnosis is made by excisional biopsy.

Given the midline location, differential diagnosis includes thyroglossal duct cyst. In contrast to thyroglossal duct cysts, dermoid cysts are smaller in size, perfectly round, more superficial (subcutaneous, superficial to strap muscles) and do not move with swallowing. Submental localization raises specific differential diagnosis: ranula is usually developed on the buccal floor but can expand to posterior part of mylohyoid muscle. Anatomical localization and extension of the cyst should be finely analyzed in order not to ignore such lesion due to salivary retention [20]. 

#### 2.4.2. Histopathological Features

**Figure 5:**
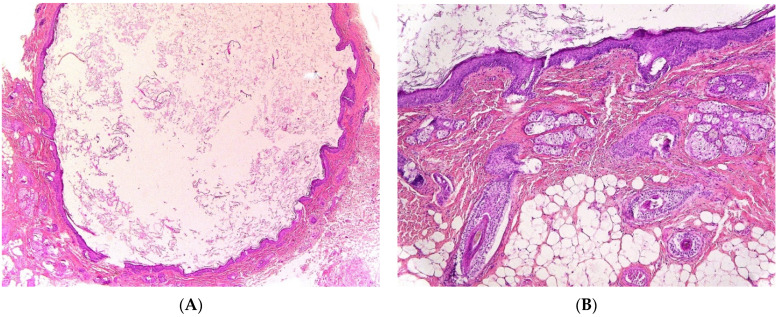

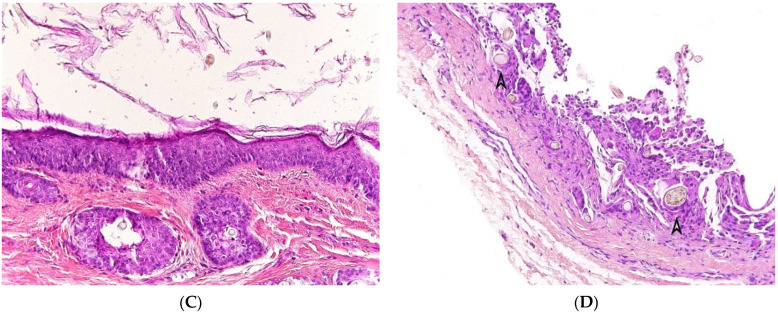
Dermoid cyst. (**A**) (H.E.S). The cyst is lined by keratinizing stratified squamous epithelium. (**B**) (H.E.S). The cyst is deeply located in the subcutis. Small hair follicles and sebaceous glands are attached to the epithelium. (**C**) (H.E.S). The lumen contains numerous vellus hair shafts and lamellated keratin, and the epithelium has a granular layer. (**D**), (H.E.S). Several vellus hairs (arrowheads) are observed within the granuloma.

At gross examination, the cyst has yellowish-white keratinous material and might reach 12 cm in size. They are lined by stratified squamous epithelium with associated small hair follicles and sebaceous glands (Figure 5A,B). Eccrine sweat glands and apocrine glands are less commonly present. The cavity contains laminated keratin and numerous vellus hairs oriented haphazardly (Figure 5C). Acute inflammation may result in the subsequent disruption of the cyst wall, with the development of an intense foreign body giant cell reaction (Figure 5D).

#### 2.4.3. Brief Account of Patient Management

The treatment of neck dermoid cysts involves complete surgical excision of the lesion [21,22].

### 2.5. Thymic Cyst

Cervical thymic cysts and ectopic thymus are extremely rare entities, with only about 100 cases reported in the literature [23]. However, the presence of asymptomatic thymic tissue in the neck is much more common, with a reported incidence of 30% in children at autopsy. They therefore represent less than 1% of cystic cervical masses. They are thought to arise due to the persistence of the embryonic thymopharyngeal ducts, derived from the ventral surface of the third pharyngeal pouch during the sixth week of intrauterine life. Failure of these ducts to regress during the eighth week of development can cause cervical thymic cysts to form.

They can arise anywhere along the descent of the thymic primordia from the mandible to the mediastinum [24]. They are most commonly found in the left neck, anterior to the sternocleidomastoid muscle, with approximately 50% showing extension into the superior mediastinum. They are most commonly present during the first decade of life, with a slight preponderance towards males.

#### 2.5.1. Clinical Findings

Cervical thymic cysts are painless neck masses that can present suddenly in children, or in association with cutaneous pharyngeal defects and ocular and facial anomalies as in branchio-oculo-facial syndrome (BOFS), an autosomal-dominant transmitted disorder with mutations in the *TFAP2A* gene. The presence of an ectopic thymus (dermal) can justify a BOFS diagnosis [25].

#### 2.5.2. Histopathological Features

**Figure 6:**
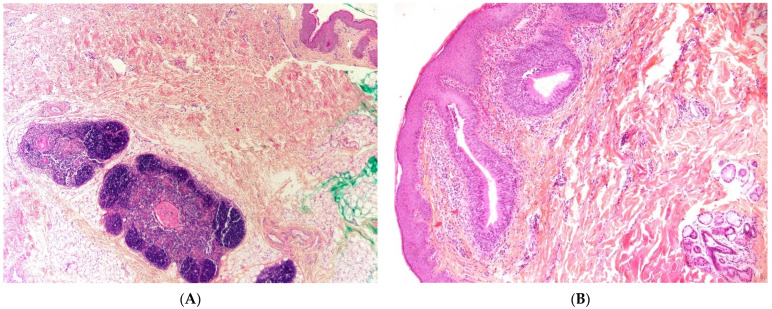
Thymic cyst. (**A**), (H.E.S). Mature thymic tissue showing Hassall’s corpuscles (arrow) and cortico-medullary differentiation and arranged in lobules at the hypodermis. The overlying dermis is devoid of adnexa and replaced by scar-like fibrous tissue. (**B**), (H.E.S). Ductal-like structures (arrow) opening at the surface of the skin, lined by a columnar, pseudostratified epithelium (at the left, half part).

The cystic fluid may be clear, yellow, brown, green, or even purulent. The diagnosis is established by histologic demonstration of residual thymic tissue showing Hassall’s corpuscles and cortico-medullary differentiation (Figure 6A). The cyst wall lining may be spindle, cuboidal, or columnar, stratified or pseudostratified, ciliated or non-ciliated (Figure 6B). Cholesterol crystals, giant cells, histiocytes, inflammatory cells, and hemosiderin have also been described.

Congenital thymic cysts should be differentiated from multilocular thymic cyst, as the latter represents an acquired, multilocular, inflammatory lesion arising from cystic dilatation of the medullary duct and associated with autoimmune diseases, such as Sjogren’s syndrome and aplastic anemia. These acquired thymic cysts are lined by squamoid, cuboidal, columnar, micropapillary or mixed glandular epithelium, and can present with pseudo-epitheliomatous hyperplasia. Secondary inflammation is commonly found but no cartilage or smooth muscle can be identified [26].

#### 2.5.3. Brief Account of Patient Management

Complete surgical excision, even with asymptomatic lesions, is used for diagnosis and treatment. Confirmation of the presence of mediastinal thymic tissue should be performed prior to the removal of thymic cysts or ectopic thymic tissue to prevent immunocompromise.

### 2.6. Bronchogenic Cyst

Bronchogenic cysts correspond to embryonic foregut abnormalities arising from aberrant budding of the tracheobronchial tree. These cysts predominantly appear in the mediastinum, especially the hilar or middle-mediastinal area. The anatomical presentation may vary, from the midline subcutaneous region of the suprasternal area to beneath the diaphragm. Cutaneous bronchogenic cysts are often supra-sternal.

#### 2.6.1. Clinical Findings

Bronchogenic cysts are often incidentally found in children and later in life. The volume usually increases with age, with the diameter ranging from 1 to 4 cm in infants. Bronchogenic cysts are usually sporadic. They are encountered predominantly within the mediastinum or the lung parenchyma and, in few instances, within the neck. Symptoms secondary to infection may occur, such as fever, hemorrhage or perforation. Bronchogenic cysts may be compressive in infants and responsible for respiratory distress. They are, however, not connected to the respiratory tract. Clinical differential diagnosis includes esophageal and enteric duplication cysts and pericardial cysts [27,28]. Diagnosis is often performed with histological analysis because of poorly specific clinical presentation: midline cyst also raise differential diagnosis as thyroglossal duct cyst, dermoid cyst, vascular malformation or thymic cervical cyst.

#### 2.6.2. Histopathological Features

**Figure 7:**
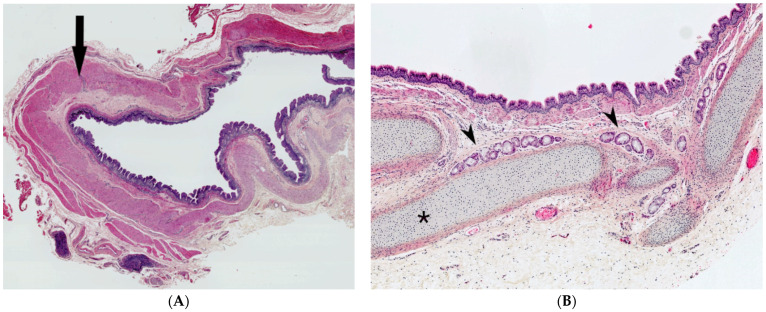
Bronchogenic cyst. (**A**), (H.E.S). unilocular cyst lined by respiratory epithelium overlying circular muscular tunic (arrow). (**B**), (H.E.S.). In the wall, seromucous glands (arrowheads) and cartilage plates (*).

The bronchogenic cyst presents itself as an extrapulmonary unilocular cyst. The lumen contains fluid, turbid, mucinous or purulent material. Its wall is lined by respiratory epithelium overlying fibromuscular connective tissue containing seromucinous glands and cartilage plates (Figure 7A,B). Squamous metaplasia may occur. The presence of cartilage tissue is important for establishing the final diagnosis and differentiating between bronchogenic cysts and other cysts of the neck [27,29].

#### 2.6.3. Brief Account of Patient Management

Surgical excision is usually necessary to confirm the diagnosis and avoid infection or hemorrhage. Bronchogenic cysts have no malignant potential.

### 2.7. Risk of Malignancy

Rare cases of malignant transformations are described at the level of congenital cystic formations. The risk of carcinomatous degeneration of the thyroglossal tract cyst is less than 1%, most often affecting the adults. Of the 180 cases reported in the literature, 21 concerned children under 18 years of age with only one case in a 6-year-old child. Most of these lesions were histologic findings following cyst removal surgery with a pathologic diagnosis of papillary carcinoma [30]. Carcinomas developed on congenital branchial cysts are also described. The reported cases concern adults and the distinction between primary carcinoma developed on an underlying congenital branchial cyst and cystic metastasis from oropharyngeal cancer remains controversial [31,32,33]. 

We would like to highlight the role of initial clinical and radiological evaluation in order not to miss malignant lesions. Radiological heterogenous aspect with even a minor solid component associated to cystic component should raise attention and lead to the realization of a biopsy. Malignant tumors may be misleading and neuroblastoma, yolk sac tumor or embryonal rhabdomyosarcoma can present with partial cystic component.

## 3. Conclusions

When evaluating a cystic lesion of the neck in children, understanding the morphologic embryology, anatomy, clinical features, and gross/histopathologic features is critical to diagnosis.

The diagnosis of a cervical mass in a child is usually suspected based on clinical examination data. The age of the child (infant or child) and the localization of the mass (median, lateral or parotid) are extremely important elements of orientation. The relative frequency of different types of cervical cysts has also to be considered. Branchial cleft cysts are the most frequent cervical cysts and represent approximately 20% of all cervical masses in children.

Ultrasound, an easy-to-perform, non-irradiating examination that does not require sedation, is a supplement; it clarifies the anatomical situation, differentiates cystic transsonic lesions from solid echogenic lesions. Most of the time, clinical examination and US are sufficient for clear identification and correct treatment of the cervical cyst.

Large predominance of benign cystic lesions does not rule out the possibility of any rare malignancies associated with a cystic presentation. These tumors have a solid component which must not be ignored on radiological examination. These situations account for rare indications of fine needle aspiration or needle biopsy in order to perform right treatment.

Finally, correct identification of these benign lesions may have additional interest in identifying or implementing the description of a genetic predisposing syndrome or chromosomal defect. Lymphatic vascular malformations, for example, may be associated with chromosomal defects.

Research on the molecular embryology of craniofacial development in the coming years should begin to shed light on the pathogenesis of lesions and syndromes of craniofacial development.

## Figures and Tables

**Table 1:** Congenital cystic masses (CCM) of the neck: frequency, location, clinical and radiologic presentation, associated syndromes and treatment.

Types of Cyst	Frequency	Usual Location	Most Common Clinical Features	Imaging Presentation	Associated Syndromes	Treatment
First branchial cleft	Second most common branchial cleft anomaly (4 to 10% of branchial cysts)	Type I, periauricular Type II, periparotid or submandibular area	Otalgia, otorrhea, parotidis, recurrent abscess at the angle of the mandible or submandibular area	CT and MRI: - superficial/deep to the parotid gland cyst - with/without EAC communication	None	Complete surgical excision
Second branchial cleft	Most common branchial cleft anomaly (80 to 95% of branchial cysts)	Lateral neck anterior to sternocleido-mastoid	Fluctuant neck mass +/− pain (when upper respiratory tract infection occurs)	Unilocular cyst, well circumscribed, homogeneous	- Branchio-oto-renal - Branchio-oculo-facial	Complete surgical excision
Third/Fourth branchial cleft	Rare (1 to 8% of branchial cysts)	Left sided predominance (97%), lower anterior neck, can be in close association to thyroid gland	Slight female preponderance Lower neck lateral cyst May present as thyroiditis	Unilocular cyst	None	Complete surgical excision
Thyro glossal duct	First common midline cyst (70% of midline CCM)	Midline fluctuant neck mass, in close relation to the hyoid bone	Asymptomatic palpable midline neck mass with movement during swallowing or tongue protrusion Pain if infected, dyspnea and/or dysphagia if large	Juxta/infrahyoid cyst that may cross the hyoid bone	Cowden	Sistrunk procedure
Dermoid	Second common midline neck cyst (25% of midline CCM)	Involving the neck: midline neck mass Involving the face: nasal or palpebral region	Midline neck mass, may be associated with a tuft of hair	MRI: variable signal intensity based on their protein/lipid/keratin content	Gardner	Complete surgical excision
Thymic	Rare 2 to 13y (1% of CCM) More common in males	Left neck, anterio-lateral with/without extension into the mediastinum	- Asymptomatic or - Upper respiratory infection, hoarseness, wheezing, coughing, dysphagia, and even respiratory distress	- Elongated, unilocular cyst - MRI > CT scan	- Branchio-oculo-facial	Complete surgical excision
Broncho-genic	Very rare More common in males	Suprasternal notch or supraclavicular area	Asymptomatic swelling	- Tubular pattern anterior to the trachea - Air within the lesion if infected	None	Complete surgical excision
Lymphatic mal-formation	Very common (20% of pediatric cervical masses)	Commonly in the posterior triangle or in the submandibular space	- Soft, painless and compressible mass, - Growing slowly, quickly if secondary to hemorrhage or infection	- Multilocular predominantly cystic mass - With septa of variable thickness	- Turner - Noonan - Trisomy 13, 18, 21 - Cowchock Wapner Kurtz	- Steroids, antibiotics - Laser, sclero-therapy, radio-frequency ablation - Surgical excision

EAC, external auditory canal; y, years; CCM, congenital cystic mass.

**Table 2:** Congenital cystic masses of the neck: gross, histologic examination and differential diagnoses.

Types of Cyst	Histologic Examination	Differential Diagnosis
First branchial cleft	- Type 1, lined by squamous epithelium - Type 2, containing skin adnexa and cartilage - Lymphoid tissue absent, unless inflammation or infection	- Dermoid cyst - Epidermic cyst - Teratoma
Second branchial cleft	- Lined by squamous epithelium (90%) or ciliated columnar epithelium (8%) and rarely by both types of epithelium (2%) - Occasionally present: salivary tissue, sebaceous glands, and cholesterol clefts with a foreign body reaction - Nodular or diffuse lymphoid tissue.	- Epidermic cyst - Dermoid cyst - Teratoma - Bronchogenic cyst - Lymphoepithelial cyst - Lymphadenitis - Lymphoma
Third/Fourth branchial cleft	- Lined by squamous epithelium or ciliated columnar epithelium and rarely by both types of epithelium - Lymphoid infiltrate - Thymic tissue - Parathyroid and thyroid gland	
Thyro-glossal duct	- Unilocular/multilocular - Lined by stratified squamous epithelium at upper part; ciliated pseudostratified columnar epithelium at lower part; Stratified cuboidal epithelium at level of hyoid bone. - Mucous glands (salivary-type). - Ectopic thyroid follicles along the course of the duct in up to 62% of cases, ectopic parathyroid; adnexal skin structures, cartilage. - Denuded epithelium and secondary inflammation (lymphocytes, neutrophils, granulation tissue, cholesterol granuloma and fibrosis).	- Epidermic cyst - Dermoid cyst - Ectopic thyroid - Teratoma - Branchial cleft cyst - Bronchogenic cyst - Lymphoepithelial cyst - Lymph node metastasis of papillary thyroid carcinoma with cystic degeneration
Dermoid	- Unilocular - Lined by keratinized squamous epithelium with a granular layer, filled with lamellate keratin. - In case of dermoid cyst, hair follicles and sebaceous glands, eccrine sweat glands in 35%, apocrine glands in 15%, occasionally smooth muscle	Sebaceous cyst Dermoid teratoma Follicular cyst Steatocystoma Vellus hair cyst
Thymic	- Unilocular - Thin wall with a few layers of bland squamoid or cuboidal cells - Thymic tissue in wall	- Acquired thymic cyst (Sjögren) - Lymphatic vascular malformation - Thyroid tumors
Broncho-genic	- Unilocular or multilocular - Lined by columnar, ciliated, pseudostratified epithelial. Squamous metaplasia in previously infected cyst - Blood vessels, hyaline cartilage, smooth muscles, sero-mucinous glands, and elastic fibers in the cyst wall.	- Branchial cysts - Thyroglossal duct cyst - Teratoma - Tracheal diverticulum
